# Robot Task-Constrained Optimization and Adaptation with Probabilistic Movement Primitives

**DOI:** 10.3390/biomimetics9120738

**Published:** 2024-12-03

**Authors:** Guanwen Ding, Xizhe Zang, Xuehe Zhang, Changle Li, Yanhe Zhu, Jie Zhao

**Affiliations:** State Key Laboratory of Robotics and System, Harbin Institute of Technology, Harbin 150001, China; 17b908042@stu.hit.edu.cn (G.D.); zangxizhe@hit.edu.cn (X.Z.); yhzhu@hit.edu.cn (Y.Z.); jzhao@hit.edu.cn (J.Z.)

**Keywords:** human–robot skill transfer, learning from demonstration, probabilistic movement primitives, task-constrained optimization, movement adaptation

## Abstract

Enabling a robot to learn skills from a human and adapt to different task scenarios will enable the use of robots in manufacturing to improve efficiency. Movement Primitives (MPs) are prominent tools for encoding skills. This paper investigates how to learn MPs from a small number of human demonstrations and adapt to different task constraints, including waypoints, joint limits, virtual walls, and obstacles. Probabilistic Movement Primitives (ProMPs) model movements with distributions, thus providing the robot with additional freedom for task execution. We provide the robot with three modes to move, with only one human demonstration required for each mode. We propose an improved via-point generalization method to generalize smooth trajectories with encoded ProMPs. In addition, we present an effective task-constrained optimization method that incorporates all task constraints analytically into a probabilistic framework. We separate ProMPs as Gaussians at each timestep and minimize Kullback–Leibler (KL) divergence, with a gradient ascent–descent algorithm performed to obtain optimized ProMPs. Given optimized ProMPs, we outline a unified robot movement adaptation method for extending from a single obstacle to multiple obstacles. We validated our approach with a 7-DOF Xarm robot using a series of movement adaptation experiments.

## 1. Introduction

With modern technological advancements, artificial intelligence (AI) is playing a pivotal role in transforming various domains, including smart cities and industrial manufacturing. In smart cities, AI, coupled with the Internet of Things (IoT) and blockchain, enables data-driven decision-making, real-time monitoring, and secure data sharing. These capabilities empower smart cities to optimize resource allocation, enhance public safety, improve traffic management, and provide personalized services to residents [1,2]. By analyzing vast amounts of data collected from sensors, IoT devices, and other sources, AI facilitates intelligent automation, helping urban systems address complex challenges efficiently [3,4].

Similarly, in industrial manufacturing, robotics is undergoing a parallel transformation driven by the need for adaptability and automation [5]. Traditionally, robotic tasks in manufacturing have been performed in carefully structured environments with repetitive trajectories. However, when the environment changes, robots often require extensive reprogramming to adapt. Addressing this limitation, AI-driven approaches, such as learning from human demonstrations, provide robots with the ability to adapt to varying task scenarios, making them more versatile and resilient [6]. In this context, Movement Primitives (MPs) emerge as a well-established method in robotics. MPs enable the generalization of movements through temporal and spatial modulation, allowing robots to sequence and combine different primitives to accomplish complex tasks efficiently [7].

The core challenge lies in how to learn and adapt MPs from finite human demonstrations. Current methods can be categorized into two groups: (i) probabilistic approaches and (ii) dynamical systems. Probabilistic approaches commonly involve methods such as Hidden Markov Models–Gaussian Mixture Regression (HMM-GMR) [8] and Gaussian Mixture Models–Gaussian Mixture Regression (GMM-GMR) [9], which model the joint probability distributions of multiple demonstrations and derive the desired trajectories via regression. Although these methods capture the statistical variability of demonstrations, they struggle to adapt to new task scenarios. To address this, Task-Parameterized Gaussian Mixture Models (TP-GMMs) [10] incorporate task parameters into GMMs, enabling adaptation to novel conditions. However, TP-GMMs fail when the task parameters are not included in the learning process. Probabilistic Movement Primitives (ProMPs) [11] represent movements via hierarchical Bayesian models with probabilistic conditioning to achieve via-point generalization, capturing correlations from different demonstrations. However, ProMPs need a great number of demonstrations to obtain reliable distributions, and the extrapolation capability is limited once via-points are out of the original range.

On the other hand, dynamical systems approaches, such as Dynamic Movement Primitives (DMPs) [12,13], describe movements using nonlinear differential equations modulated by external forcing terms. DMPs are well suited for generalizing start and end points from a single demonstration but are unable to handle intermediate via-points effectively. To enhance their capabilities, some works have combined DMPs with reinforcement learning (RL) to learn couplings across different control variables [14].

Recently, hybrid methods that combine DMPs and ProMPs have been proposed. Probabilistic Dynamic Movement Primitives (ProDMPs) [15] convert DMPs’ numerical integration into basis functions to denote trajectories with ProMPs. However, the number of DMP trajectories required to learn ProMPs affects the performance. Via-point Movement Primitives (VMPs) [16] denote movements with elementary and shape trajectories, adapting to single or multiple demonstrations. A threshold is defined to distinguish DMP extrapolation and ProMP interpolation. However, VMPs may result in high accelerations at transition points with nonsmooth trajectories.

When adapting MPs to different task scenarios, via-point generalization is not sufficient. Task constraints manifested as inequalities like position and velocity limits, obstacles, and virtual walls are imposed on MPs. The objective is to generate trajectories in close proximity to the original trajectories while satisfying task constraints. MP adaptation methods are extensively researched; for instance, DMPs extend obstacle avoidance by imposing repulsive forces in the acceleration domain [17]. Position limits are ensured by transforming trajectories into exogenous states [18]. However, DMPs are prone to becoming trapped in local minima when multiple forces act mutually. Moreover, DMPs are adapted to a particular trajectory, losing additional freedom to execute tasks in different ways.

In contrast, ProMPs encode variations with a covariance matrix, and the variations can be further exploited to satisfy task constraints. Ref. [19] combines multiple ProMPs to achieve obstacle avoidance. However, it is impractical to foresee all situations by adding primitives. Another way is to exclude specific regions from the original ProMPs via optimization; for instance, ref. [20] formulates optimization as a policy search problem with Kullback–Leibler (KL) divergence and reward functions defined to derive the policy, while the work in [21] frames optimization by minimizing the Mahalanobis distance to the original ProMPs in conjunction with obstacle distance computation. However, the methods mentioned above only optimize ProMPs for the robot end-effector, with no regard to possible collisions for other robot links. Moreover, they only handle obstacle constraints, and other task constraints, like virtual walls and joint limits, are not considered. Ref. [22] incorporates multiple task constraints into an integrated probabilistic framework; they directly optimize ProMPs with gradient ascent–descent algorithms. However, ProMPs will affect Gaussians in each timestep, and the timesteps satisfying task constraints will be optimized, leading to a long optimization time.

In this work, we propose an improved via-point generalization method that allows a robot to acquire skills from only one demonstration. ProMPs are encoded by generalizing trajectories with via-points rather than providing more demonstrations. Furthermore, we propose an improved task-constrained ProMP adaptation method by separating each timestep with Gaussians and only optimizing feature timesteps violating task constraints.

To summarize, our contributions are as follows:

(1) We propose an improved via-point generalization method that allows a robot to acquire skills from only one human demonstration with smooth trajectories generalized to learn ProMPs;

(2) We propose an effective task-constrained ProMP optimization method that incorporates all task constraints analytically into a probabilistic framework;

(3) Given optimized ProMPs, we propose a unified robot movement adaptation method extending from a single obstacle to various obstacles.

This paper is organized as follows: Section 2 provides derivations of the theoretical formulas and methods involved in this paper, with Section 2.1 presenting via-point trajectory generalization and ProMP encoding, Section 2.2 formulating the task-constrained optimization problem, and Section 2.3 introducing the optimization procedure; Section 3.2 outlines the complete robot movement adaptation procedure in principle. We validate and compare our method through experiments in Section 4. Finally, we conclude this work and outline future research directions. For clarity, all definitions and notations employed in this paper are summarized in Table 1.

## 2. Theoretical Formulation and Methodology

### 2.1. Generating Trajectories and Encoding Distributions

#### 2.1.1. Via-Point Trajectory Generalization

**Figure 1:**
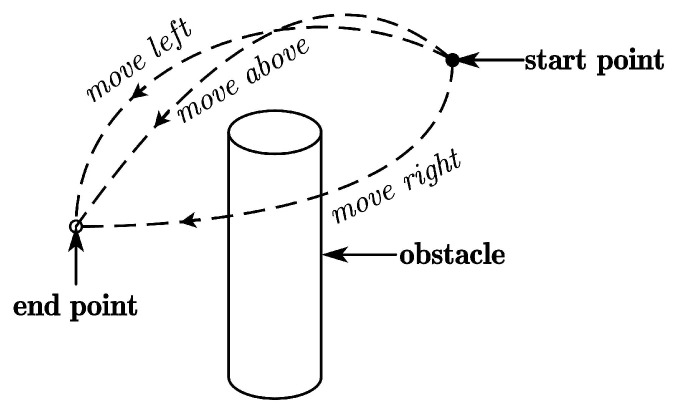
Three skills for a robot to move around an obstacle based on human demonstration.

Via-point trajectory generalization enables robots to quickly generate a diverse set of feasible motions for a given skill while retaining key features from human demonstrations. This process includes generalizing both positions and orientations, allowing the robot to efficiently generate task-specific trajectories while maintaining task diversity by teaching the robot new skills. We provide three modes, namely, moving above, left, and right around the obstacle, as shown in Figure 1. Only one demonstration is required for each mode, and it is obtained by tracking human hand movements with the Kinect FORTH system [23]. We select the human hand centroid as the tracking point, with position and orientation sets $\left\{x,y,z,q_{x},q_{y},q_{z},q_{w}\right\}$ recorded in *T* timesteps. To ensure trajectory smoothness, we apply a Moving Average Filter (MAF) with a window width *n* and transform the trajectory into the robot base frame. To learn and generalize human demonstration features, we express the trajectory as a combination of the elementary trajectory *h* and shape modulation *f*.

(1) Position Generalization

Regarding 3D positions, we denote position sets p by $y\left(p\right)=h\left(p\right)+f\left(p\right)$. The elementary trajectory $h\left(p\right)$ is a line directly connecting the start point $p_{1}$ and the end point $p_{end}$, with the shape modulation defined as $f\left(p\right)=p-\left(p_{1}+r\vec{p_{1}p_{\mathrm{end}}}\right)$, where r is the scale factor denoting the proportion of projective points on the line $h\left(p\right)$. If no intermediate via-points are required, we directly generate new projective points between a new start point $p_{1}^{\mathrm{new}}$ and end point $p_{\mathrm{end}}^{\mathrm{new}}$ according to r, with the learned shape modulation $f\left(p\right)$ added to generalize new trajectory sets $p^{\mathrm{new}}=p_{1}^{\mathrm{new}}+r\vec{p_{1}^{\mathrm{new}}p_{\mathrm{end}}^{\mathrm{new}}}+f\left(p\right)$. Once a via-point $\left(t_{\mathrm{via}},p_{\mathrm{via}}\right)$ is needed, we scale the shape modulation with the coefficient $k=\left|p_{\mathrm{via}}-\left(p_{1}^{\mathrm{new}}+r\vec{p_{1}^{\mathrm{new}}p_{\mathrm{end}}^{\mathrm{new}}}\right)\right|{\left|f\left(p\right)\right|}^{-1}$. Subsequently, new trajectory sets are generalized to $p^{\mathrm{new}}=p_{1}^{\mathrm{new}}+r\vec{p_{1}^{\mathrm{new}}p_{\mathrm{end}}^{\mathrm{new}}}+kf\left(p\right)$.

(2) Orientation Generalization

Considering that orientation dimensions are affected by each other, we define the trajectory as $y\left(o\right)=h\left(o\right)\times f\left(o\right)$, where $o=\left[q_{w},q_{x},q_{y},q_{z}\right]$ represents quaternion sets, and × denotes quaternion multiplication. Spherical linear interpolation (SLERP) provides an efficient way to interpolate between two quaternions while preserving their spherical geometry. It ensures a constant angular velocity along the shortest path on the unit sphere, which results in smooth and natural transitions between orientations. We thus derive the elementary trajectory $h\left(o\right)$ by SLERP with the ratio set uniformly from 0 to 1 to learn $f\left(o\right)$. Once new start and end quaternion sets are required during generalization, we compute a new elementary trajectory $h{\left(o\right)}^{\mathrm{new}}$ with the learned shape modulation $f\left(o\right)$ superimposed to generate a new orientation trajectory $y{\left(o\right)}^{\mathrm{new}}$. Note that there is no need to generalize orientation via-points, as we can generalize 3D positions with via-points while keeping the original orientations.

#### 2.1.2. Modeling Joint Distributions with Probabilistic Movement Primitives

Probabilistic Movement Primitives (ProMPs) [24] are probabilistic methods for encoding variances in joint or Cartesian space. To integrate all task constraints analytically, we encode ProMPs in joint space. Given the position and orientation sets generalized in Section 2.1.1, we obtain all joint sets ${\left\{q_{t}\right\}}_{i=1}^{N}$ via the Jacobian inverse kinematics (IK) algorithm, where $q_{t}\in R^{D}$, $t\in \left[0,T\right]$, *N* is the demonstration number, and *D* is the DoF number. ProMPs model joint positions as a linear combination of Gaussian basis functions $\Psi \left(t\right)$ and a weight vector $w_{i}$ in the presence of zero-mean Gaussian noise as $\varepsilon_{n}$, $q_{i}\left(t\right)=\Psi {\left(t\right)}^{T}w_{i}+\varepsilon_{n}$. Joint positions in one demonstration are expressed with the weight vector $w_{i}$ via linear ridge regression [25]. Using the parameters $\theta =\left\{\mu_{w},\Sigma_{w}\right\}$ to estimate the weight vector w with Gaussian distributions, the probability of observing a trajectory τ given θ becomes

(1) $$\begin{array}{cc} \; & p\left(\tau ;\theta \right)=p\left(\tau |w\right)p\left(w;\theta \right)dw\\ \; & =\prod_{t}N\left(\left.q\left(t\right)\right|\Psi {\left(t\right)}^{T}\mu_{w},\Psi {\left(t\right)}^{T}\Sigma_{w}\Psi \left(t\right)+\Sigma_{n}\right) \end{array}$$

where $\mu_{w}$ and $\Sigma_{w}$ denote the mean and covariance of the weight vector, which are derived by maximum likelihood estimation, and $\Sigma_{n}$ is the Gaussian noise covariance. To model joint distributions with ProMPs, we first transform the human hand trajectory into the robot base frame and derive the corresponding joint values. These joint values are then represented as a combination of *K* Gaussian basis functions and their associated weights. We compute the mean and covariance of the weights. The weight distributions capture the variability in human demonstrations and enable the robot to execute tasks flexibly. During task execution, the robot samples trajectories from the joint distribution at each timestep, ensuring adaptability while preserving the demonstrated variability.

### 2.2. Optimizing Distributions with Task Constraints

#### 2.2.1. Problem Formulation

Given the initial ProMPs $p_{0}\left(w\right)=N\left(w;\mu_{w}^{0},\Sigma_{w}^{0}\right)$, the main objective is to obtain optimized ProMPs that are as close as possible to the original ProMPs while satisfying task constraints. Achieving this trade-off requires a method to quantify the similarity between distributions while effectively encoding task constraints. In this context, KL divergence is particularly suitable due to its closed-form solution for Gaussian distributions, ensuring computational efficiency. Additionally, the cumulative distribution function (CDF) offers a probabilistic framework that incorporates variability and uncertainty into the constraints, considering the stochastic nature of ProMPs. As a result, we use KL divergence to denote the similarity and formulate task constraints with a cumulative distribution function (CDF). Since ProMPs model each timestep as a Gaussian distribution, we separate each timestep with Gaussian distributions by converting the initial ProMPs to the mean and standard deviation $\left(\mu_{t}^{0},\sigma_{t}^{0}\right)$ of each timestep *t*, with the new mean and standard deviation $\left(\mu_{t}^{*},\sigma_{t}^{*}\right)$ optimized under task constraints separately. Let $c_{k,t}=C\left(\mu_{t},\sigma_{t}\right)$ denote the $k^{th}$ task constraint function related to $\left(\mu_{t},\sigma_{t}\right)$, and let $F_{c_{k,t}}\left(\mu_{t},\sigma_{t}\right)$ denote the CDF of $c_{k,t}$. We can rewrite the optimization problem as a Lagrangian function:

(2) $$\begin{array}{cc} L\left(\mu_{t},\sigma_{t},\left\{\lambda_{k,t}\right\}\right)= & D_{\mathrm{KL}}\left[N\left(q_{t};\mu_{t},\sigma_{t}\right)\left|\right|N\left(q_{t}^{0};\mu_{t}^{0},\sigma_{t}^{0}\right)\right]\\ \; & +\sum_{k,t}\lambda_{k,t}\left[\alpha_{k,t}-F_{c_{k,t}}\left(\mu_{t},\sigma_{t}\right)\right] \end{array}$$

where $\alpha_{k,t}$ is the confidence level to represent the task constraint probability $P_{\mu_{t},\sigma_{t}}\left(H\left(c_{k,t}\right)\right)⩾\alpha_{k,t}$, *H* represents one-sided or two-sided inequality constraints, and the optimized $\left(\mu_{t}^{*},\sigma_{t}^{*}\right)$ are derived by gradient ascent–descent methods: $\left(\mu_{t}^{*},\sigma_{t}^{*},\left\{\lambda_{k,t}^{*}\right\}\right)=\max_{\lambda_{k,t}⩾0}\;\;\min_{\mu_{t},\sigma_{t}}\;\;L\left(\mu_{t},\sigma_{t},\left\{\lambda_{k,t}\right\}\right)$. Subsequently, given $\left(\mu_{t}^{*},\sigma_{t}^{*}\right)$, we implement another optimization step to obtain the optimized ProMPs as $\mu_{w}^{*}=\min_{\mu_{w}}\left(\Psi_{t}^{T}\mu_{w}-\mu_{t}^{*}\right),\Sigma_{w}^{*}=\min_{\Sigma_{w}}\left(\Psi_{t}^{T}\Sigma_{w}\Psi_{t}-\sigma_{t}^{*}\right)$. In the next section, we formulate every task constraint by defining the function $c_{k,t}=C\left(\mu_{t},\sigma_{t}\right)$ related to $\left(\mu_{t},\sigma_{t}\right)$, expressing the inequality constraint function *H* and approximating the CDF $F_{c_{k,t}}\left(\mu_{t},\sigma_{t}\right)$.

#### 2.2.2. Constraint Definitions in Joint and Cartesian Space

(1) Joint Range Limit

In this paper, we focus on retaining the original joint ranges learned from ProMPs. We formulate the constraint function $c_{k,t}=q_{t}\left(\mu_{t},\sigma_{t}\right)$ and denote two-sided constraints by $P_{\mu_{t},\sigma_{t}}\left(q_{k,t}^{\min}<q_{t}\left(\mu_{t},\sigma_{t}\right)<q_{k,t}^{\max}\right)⩾\alpha_{k,t}$, where *k* denotes the $k^{th}$ joint’s freedom, $\alpha_{k,t}$ is the confidence level, and $q_{k,t}^{\min}$ and $q_{k,t}^{\max}$ are the minimum and maximum joint positions. We approximate joint positions as Gaussians and calculate the difference in the CDF to obtain $F_{c_{k,t}}\left(\mu_{t},\sigma_{t}\right)$.

(2) Waypoints

Waypoints that we expect the robot to reach at timestep *t* are defined in the Cartesian space with the FK function *T*. Based on the distance square between expected and actual points, we denote the waypoint constraint function by $c_{k,t}={\left|T\left(q_{t}\left(\mu_{t},\sigma_{t}\right)\right)-{\bar{X}}_{t}\right|}^{2}$ and express the one-sided constraint as $P_{\mu_{t},\sigma_{t}}\left(|T\left(q_{t}\left(\mu_{t},\sigma_{t}\right)\right)-{\bar{X}}_{t}{|}^{2}⩽d_{k,t}^{2}\right)⩾\alpha_{k,t}$, where $d_{k,t}$ is the distance threshold, and $\alpha_{k,t}$ is the confidence level. Because of the nonlinear nature of $c_{k,t}$, we estimate the distribution as follows:

(3) $$\begin{array}{cc} S=\{c_{k,t}\left(q_{1},q_{2},...,q_{D}\right),q_{k} & \in \{\mu_{k}-2\sigma_{k},\mu_{k}+2\sigma_{k}\}\}\\ E\left(c_{k,t}\right)\approx & \mathrm{Mean}\left[S\right]\\ V\left(c_{k,t}\right)\approx & \mathrm{Var}\left[S\right] \end{array}$$

where *D* is the DOF number, and we utilize 2x the standard deviation to constitute the set *S* and compute the mean and variance with moment matching. The random variable $c_{k,t}$ distribution is a generalized $\chi^{2}$, and we approximate it with a simpler Gamma distribution with shape α and rate β to represent the CDF analytically. The CDF is then computed with $F_{c_{k,t}}=F\left(d_{k,t};\Gamma \left(\alpha ,\beta \right)\right)$ to denote the waypoint constraint probability.

(3) Hyperplane

Hyperplane constraints, known as virtual walls, are defined in Cartesian space to restrict the robot to specific planes. We formulate the hyperplane constraint function as $c_{k,t}=n_{k,t}^{T}\left(T\left(q_{t}\left(\mu_{t},\sigma_{t}\right)\right)-b_{k,t}\right)$, where $n_{k,t}$ and $b_{k,t}$ are the hyperplane normal and bias vectors, with a one-sided inequality constraint expressed as $P_{\mu_{t},\sigma_{t}}\left(n_{k,t}^{T}\left(T\left(q_{t}\left(\mu_{t},\sigma_{t}\right)\right)-b_{k,t}\right)⩽0\right)⩾\alpha_{k,t}$. The $c_{k,t}$ distribution is a linear transformation of Cartesian 3D positions $T\left(q_{t}\left(\mu_{t},\sigma_{t}\right)\right)$; however, *T* is nonlinear in $\left(\mu_{t},\sigma_{t}\right)$. We thus use our moment-matching method (Equation (3)) to obtain the estimated mean $E\left[T\right]$ and variance $V\left[T\right]$, so the overall distribution becomes $c_{k,t}∼N\left(\begin{array}{c} n_{k,t}^{T}\left(E\left[T\right]\right)-b_{k,t}\;\;,n_{k,t}^{T}V\left[T\right]n_{k,t} \end{array}\right)$, with the CDF computed with Gaussians to denote the probability.

(4) Repellers

Repeller constraints between the robot and obstacles are imposed on the robot end-effector or other links. We define the danger distance considering only dangerous situations where the robot is prone to collide. We compute the shortest distance between the robot link and obstacles with the minimum distance $d_{\min}$ found. If $d_{\min}$ is greater than the obstacle safety radius $R_{\mathrm{safe}}$, the danger distance is 0; otherwise, the danger distance is $d_{\mathrm{danger}}=R_{\mathrm{safe}}-d_{\min}$. We formulate the constraint function and express the one-sided inequality to show the probability of $d_{\min}⩾R_{\mathrm{safe}}$ as $P_{\mu_{t},\sigma_{t}}\left(d_{danger}⩽0\right)⩾\alpha_{k,t}$. The $c_{k,t}$ distribution is nonlinear in $\left(\mu_{t},\sigma_{t}\right)$; we thus utilize Equation (3) to estimate and compute the CDF with Gaussians to denote the probability.

### 2.3. Optimization Procedure

Based on the Lagrangian function and constraint function defined in Section 2.2.2, we concretize the procedure to obtain optimized parameters. For notational brevity, we use the notations $\theta_{0}=\left(\mu_{t}^{0},\sigma_{t}^{0}\right)$ and $\theta =\left(\mu_{t},\sigma_{t}\right)$ to denote the initial parameters and optimized parameters. We rewrite the Lagrangian function in Equation (2):

(4) $$L\left(\theta ,\left\{\lambda_{k,t}\right\}\right)=D_{\mathrm{KL}}\left[\theta ||\theta_{0}\right]+\sum_{k,t}\lambda_{k,t}\left(\alpha_{k,t}-F_{c_{k,t}}\left(\theta \right)\right)$$

The term $D_{\mathrm{KL}}\left[\theta ||\theta_{0}\right]$ denotes the KL divergence between two univariate Gaussian distributions, with the optimized parameters derived as $\left(\theta^{*},\left\{\lambda_{k,t}^{*}\right\}\right)=\max_{\lambda_{k,t}⩾0}\;\;\min_{\theta }\;\;L\left(\theta ,\left\{\lambda_{k,t}\right\}\right)$. We employ a double-loop gradient ascent–descent algorithm because it is well suited for solving optimization problems involving both maximization and minimization objectives. In this context, the outer loop optimizes the Lagrange multipliers $\lambda_{k,t}$, which enforce the task constraints, while the inner loop minimizes the parameters θ to achieve the best fit to the desired ProMPs. This approach ensures efficient convergence by decoupling the updates of the dual and primal variables, enabling the algorithm to effectively handle the inherent coupling between task constraints and distribution similarity. The double-loop structure also provides a systematic way to balance the trade-off between satisfying the constraints and minimizing the KL divergence. To be specific, for the outer loop, we optimize $\lambda_{k,t}$ with gradient quasi-ascent steps. We use the Exponential Method of Multipliers (EMM) [26] to solve inequality constraints via exponential factors by expressing the optimized $\lambda_{k,t}$ after each iteration as $\lambda_{k,t}^{\left(s+1\right)}=\lambda_{k,t}^{\left(s\right)}.\exp\left\{\eta_{k}\partial_{\lambda_{k,t}}L\right\}$, where $\eta_{k}$ decides the convergence speed, and the gradient of $\left\{\lambda_{k,t}\right\}$ is obtained by $\partial_{\lambda_{k,t}}L=\alpha_{k,t}-F_{c_{k,t}}\left(\theta \right)$. For the inner loop, we optimize θ with the gradient descent method. We adopt the LBFGS [27] method to conduct nonlinear optimization with low memory cost. We perform several LBFGS steps, with the gradient $\partial_{\theta }L$ computed using the Tensorflow automatic differentiation framework [28]. The convergence conditions are checked after each iteration to speed up the process. Given the optimized $\left(\mu_{t}^{*},\sigma_{t}^{*}\right)$, we perform several LBFGS steps to obtain $\left(\mu_{w}^{*},\Sigma_{w}^{*}\right)$. For parameter selection, we start by choosing initial values for the parameters $\theta_{0},\left\{\lambda_{k,t}^{0}\right\},\eta_{k}$ and then iteratively update them through inner- and outer-loop optimizations until convergence. To integrate the methods presented in this section, the overall optimization procedure is summarized in Algorithm 1.
**Algorithm 1** Task-constrained optimization algorithm of ProMPs.**Input:** $\left(\mu_{w}^{0},\Sigma_{w}^{0}\right)$⊳ Original ProMPs**Output:** $\left(\mu_{w}^{*},\Sigma_{w}^{*}\right)$⊳ Optimized ProMPs**Initialization:**• Compute $\left(\mu_{t}^{0},\sigma_{t}^{0}\right)$.• Derive $c_{k,t}$, *H*, and $F_{c_{k,t}}\left(\mu_{t},\sigma_{t}\right)$ (see Section 2.2.2) to construct Equation (2).•$\theta^{0}\leftarrow \theta_{0},\left\{\lambda_{k,t}^{0}\right\}$ defined in Equation (4).**□ Learnable parameters 1: **$\left(\mu_{t}^{*},\sigma_{t}^{*},\lambda_{k,t}^{*}\right)$**Optimization 1:**    **repeat**⊳ Double-loop optimization       **repeat**⊳ Descent       $\theta^{\left(s+1\right)}\leftarrow$ LBFGS $\left(L,\partial_{\theta }L,\theta^{\left(s\right)},\left\{\lambda_{k,t}^{\left(r\right)}\right\}\right)$    **until** inner-loop condition        $\lambda_{k,t}^{\left(r+1\right)}\leftarrow \lambda_{k,t}^{\left(r\right)}.\exp\left\{\eta_{k}\left(\alpha_{k,t}-F_{c_{k,t}}\left(\theta \right)\right)\right\}$⊳ Ascent**until** converged**□ Learnable parameters 2: **$\left(\mu_{w}^{*},\Sigma_{w}^{*}\right)$**Optimization 2:****repeat**    $\left(\mu_{w}^{\left(s+1\right)},\Sigma_{w}^{\left(s+1\right)}\right)\leftarrow$ LBFGS $\left(\mu_{w}^{\left(s\right)},\Sigma_{w}^{\left(s\right)},\mu_{t}^{*},\sigma_{t}^{*}\right)$**until** converged

## 3. Adapting Movement with ProMPs

### 3.1. Movement Adaptation with a Single Obstacle

Given a single obstacle, we provide three modes to move around the obstacle. We generalize trajectories to the same start and end points without via-points (see Section 2.1.1) and normalize them to the same timestep *T*, treated as the new template trajectory. To generate feasible trajectory sets that will not collide, we establish feature via-points at equal intervals within the appropriate range. We compute the distance between the robot end-effector and the obstacle and fine-tune the trajectory to ensure safety if necessary. To facilitate the distance calculation and trajectory generalization, we model the robot end-effector as a sphere. If the robot end-effector minimum *Z* is above the obstacle’s upper surface, the trajectory is safe, and if the minimum *Z* is below it, the trajectory is safe when the axial distance is greater than the total safety radius. We adapt feature via-points in ways determined by different modes to generalize a safe trajectory when the trajectory is prone to collide.

For each mode, *N* safe robot end-effector trajectories are generalized. We use Jacobian IK to obtain all feasible joint sets ${\left\{q\right\}}_{i=1}^{N}$ and encode ProMPs $p_{0}\left(w\right)=N\left(w;\mu_{w}^{0},\Sigma_{w}^{0}\right)$. To speed up optimization, we first find feature timesteps $t_{\mathrm{feature}}$ actually violating task constraints and optimize distributions using Algorithm 1. The optimized ProMPs are treated as the safe joint distributions.

During task execution, the robot first determines start and end points and then selects the mode $k∼\left\{above,left,right\right\}$ and samples via-points with Gaussians $p_{via}=N\left(\mu_{xyz},\sigma_{xyz}\right)$. The mean $\mu_{xyz}$ and standard deviation $\sigma_{xyz}$ are computed from the initial via-point range. We generalize and fine-tune the trajectory to ensure safety with sampled via-points. The optimized $\mu_{t}^{*}$ and $\sigma_{t}^{*}$ represent the joint limit range as $q_{t}^{\mathrm{safe}}\in \left(\mu_{t}^{*}-2\sigma_{t}^{*},\mu_{t}^{*}+2\sigma_{t}^{*}\right)$, and the initial joint value is denoted by $q_{t}^{0}=\Psi_{t}^{T}w^{*}$. The joint limited range and initial value are passed to the Jacobian IK algorithm to test whether the solution is calculated successfully. The success number of all feature timesteps decides whether the robot can perform the task successfully: that is, if the success number is more than a threshold, the robot directly runs the generalized trajectory; otherwise, the robot continues sampling until success. To obtain the threshold, we perform equally spaced sampling from the initial via-point range, record the success result and corresponding success number, and then determine the threshold from success to failure.

### 3.2. Movement Adaptation with Various Obstacles

Based on the assumption that we are trying to actively avoid every obstacle in the environment, we define *M* obstacles’ adaptation as a sequential combination of obstacle pairs. Consequently, we segment the trajectory with *M* segments learned with ProMPs, which are separately optimized via Algorithm 1. For each segment, we first determine the start and end points according to the two obstacles’ relative positions. When axial distances are able to accommodate the end-effector, we take the middle point between two obstacles; if not, we take the middle point above. The robot first generalizes safe trajectory sets from different modes and encodes ProMPs, then optimizes ProMPs under task constraints, and finally samples from different modes for each segment and connects all segments together for task execution. The unified movement adaptation method is presented in Algorithm 2.
**Algorithm 2** Robotic task-constrained movement adaptation algorithm.**Input:**• Obstacle information ${\left\{{\mathrm{obs}}_{m}\right\}}_{m=1}^{M}$• Mode template trajectory ${\left\{x,y,z,q_{x},q_{y},q_{z},q_{w}\right\}}_{k=1}^{3}$• Global task start point and end point $\left\{p_{1},p_{\mathrm{end}}\right\}$**Output:** Task execution trajectory $\left\{x,y,z,q_{x},q_{y},q_{z},q_{w}\right\}$□ **Generalize and Optimize with ProMPs****for** m=1 to *M* **do**   • Determine the local start point and end point (see Section 3.2) and generate *N* safe trajectories.   • Obtain feasible joint sets ${\left\{q\right\}}_{i=1}^{N}$ and encode ProMPs $\left(\mu_{w}^{0},\Sigma_{w}^{0}\right)$.   • Find feature timesteps $\left\{t_{\mathrm{feature}}\right\}$ that violate task constraints.   • Optimize ProMP distribution $\left(\mu_{w}^{*},\Sigma_{w}^{*}\right)$ using Algorithm 1.   • Obtain the optimized joint limit range and the initial joint value $q_{t}^{\mathrm{safe}}\in \left(\mu_{t}^{*}-2\sigma_{t}^{*},\mu_{t}^{*}+2\sigma_{t}^{*}\right),q_{t}^{0}=\Psi_{t}^{T}w^{*}$.   • Determine the success threshold (see Section 3.1).   **repeat**       • Sample modes $k∼\left\{above,left,right\right\}$.       • Sample via-points $p_{via}=N\left(\mu_{xyz},\sigma_{xyz}\right)$.       • Generalize and fine-tune the trajectory.       • Test the success number of $\left\{t_{\mathrm{feature}}\right\}$ via Jacobian IK   **until** success.**end for**□ **Task Execution**• Connect each segment trajectory and execute the task.

## 4. Experimental Validation

In this section, we evaluate the proposed method in an industrial scenario where the robot attempts to grasp the emergency stop while satisfying task constraints. We extended the scenario from a single obstacle to multiple obstacles and conducted comparative experiments with other methods to verify the proposed approach. After learning different skills from human demonstration, the robot was required to autonomously perform the task of grasping the emergency stop under varying task constraints.

### 4.1. Experiments with a Single Obstacle

For this study, we selected the Ufactory Xarm 7-DoF robotic arm as the experimental platform. The Kinect V2 camera is strategically positioned on the side to track human hand movements, capturing continuous centroid poses. This setup ensures that the human demonstrations are recorded accurately, even in dynamic conditions. Additionally, a Zed2i stereo camera is mounted overhead, enabling the precise acquisition of object poses, which are essential for understanding the spatial context and interaction requirements within the workspace. This dual-camera configuration offers a complementary perspective, enhancing the overall robustness of the system.

Given a single obstacle in the workspace, we provide three modes to move around the obstacle. Each mode involves the continuous tracking of human hand centroid poses, facilitated by the Kinect FORTH system, ensuring smooth and real-time trajectory acquisition. To refine the raw trajectories obtained from human demonstrations, we apply a Moving Average Filter (MAF) with n=5 to eliminate noise and ensure smooth transitions between timesteps. Subsequently, these trajectories are normalized to a uniform length of 30 timesteps, enabling consistent execution across all modes. The trajectories are then transformed into the robot base frame and further converted to the table frame defined at the tabletop surface. To guarantee executable trajectories for the robot, we offset the trajectories at the minimum point of the gripper center.

**Figure 2:**
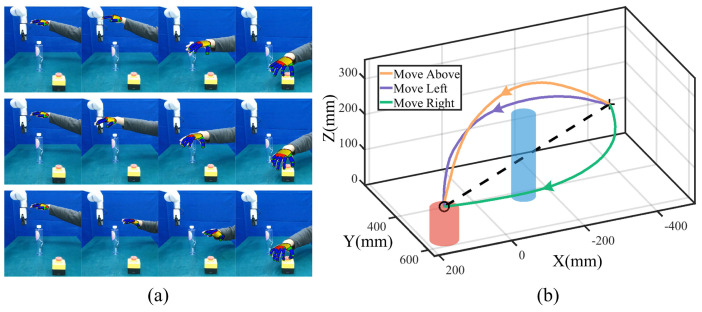
Trajectory acquisition process. (**a**) Snapshots of tracking human hand movements. The first, middle, and last rows denote moving above, left, and right modes. (**b**) Template trajectories for three modes at the same start point (symbol ‘+’) and end point (symbol ‘o’). The elementary trajectory is denoted by the black dashed line.

During task execution, the start point is fixed to ensure repeatability, while the end point is determined dynamically based on emergency stop poses detected by YOLO [29]. This integration of real-time object detection enhances the system’s safety and adaptability, allowing for robust performance in unpredictable scenarios. The trajectory acquisition process and corresponding template trajectories are shown in Figure 2a,b.

As illustrated in Figure 2a, the Kinect FORTH system demonstrates the capability to track human hand movements in real time, providing a stable and accurate estimation of the hand centroid. A colorful fingertip can quickly match the human hand’s demonstration and achieve accurate pose estimation. This stability ensures that the robot effectively acquires human skills from only one demonstration. After preprocessing, we generalize the template trajectories for three modes with the same start and end points, as shown in Figure 2b. There are no intermediate via-points for template trajectory generalization. The robot adapts to practical task conditions by dynamically adjusting to different start and end points. The generalized trajectories retain smoothness and preserve the key features of the human demonstration, resulting in robot movements that closely mimic the demonstrated skills.

After generating template trajectories for each mode, we proceed to generalize collision-free Cartesian trajectory sets for the minimum point of the gripper center. We first determine the timestep and the corresponding point on the template trajectory exhibiting the maximum deviations for each mode. The point is selected as the via-point. Subsequently, we determine the XYZ range for the via-point and adjust the range by discretizing it with equal spacing.

**Figure 3:**
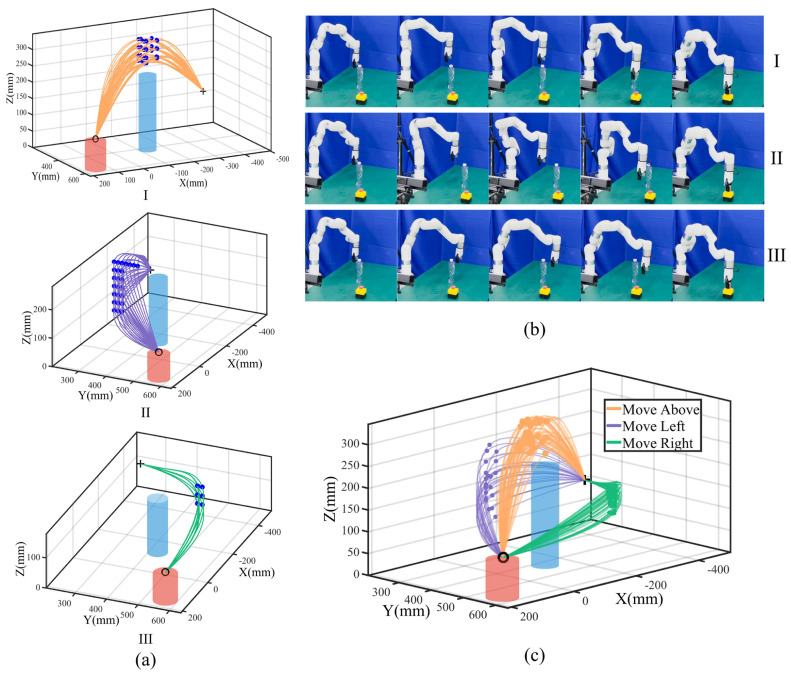
An experiment with a single obstacle. (**a**) Generalized collision-free trajectory sets. I, II, and III correspond to above, left, and right modes with blue feature via-points. (**b**) Task execution snapshots. The first, middle, and last rows show the above, left, and right modes. (**c**) Task execution trajectories with orange, purple, and green lines (points) denote trajectories (feature via-points).

For the above mode, X and Y are within ±36.23 mm (the safe obstacle radius) around the obstacle, with Z at ±100 mm around; for the left and right modes, we fix X with Y and Z at ±100 mm around. We apply a union of the ranges with the limits Y and Z for task execution. We traverse from the X, Y, and Z ranges with equal spacing to obtain feature via-point sets and generalize initial trajectory sets. We further fine-tune the trajectory based on the distance between the robot and the obstacle. The fine-tuning process involves finding the most dangerous point, which is the point closest to the obstacle, and adjusting the trajectory to ensure that this point does not enter the danger zone. For the above mode, this typically involves setting the Z-coordinate to a safe value. In the left and right modes, where the Y-coordinate plays a more crucial role in avoiding obstacles, we adjust the Y-coordinate to maintain a safe distance. These fine-tuned points are then treated as new feature via-points to refine and generalize collision-free trajectory sets for each mode shown in Figure 3a.

We further compute corresponding collision-free joint sets via Jacobian IK and encode ProMPs. We set 35 basis functions to learn the original 7-DoF ProMPs and formulate task constraints to find feature timesteps violating task constraints. The task constraints include several key factors:

(1) The joint range limit ensures that each joint remains within its specified range throughout the task, which is essential for preventing mechanical damage or errors during movement.

(2) Waypoint constraints are used to ensure the start and end points. These waypoints are defined in the task space and are crucial for ensuring that the robot reaches the desired locations accurately. The forward kinematics (FK) function *T* calculates the end-effector positions based on the joint configurations, and we set a precision requirement of $d_{k,t}=1$ mm to ensure that the task is completed with high accuracy.

(3) Hyperplane constraints define the spatial limits within which the robot’s movements are confined. For example, the maximum Z-plane is constrained to a hyperplane defined by $nz,t=\left(0,0,-1\right),bz,t=\left(0,0,575\right)$, ensuring that the robot’s end-effector stays below the table in the Z-direction. We define the hyperplane constraints for Y and Z as 100<Y<700,10<Z<575 in the experiment.

(4) Repeller constraints account for potential collisions between the robot’s links. For instance, we define repeller constraints for robot link 6 to link 1, ensuring that the robot avoids colliding with the obstacle during its motion. The obstacle in this task is a water bottle, which has a safe obstacle radius of $R_{\mathrm{safe}}=36.23$ mm.

For all task constraints, we set a confidence parameter $\alpha_{k,t}=0.95$ to account for the 2x standard deviations of uncertainty, and we use our moment-matching method to estimate nonlinear task constraints, such as waypoints, hyperplanes, and repellers. This method allows for estimating task constraints based on probabilistic models, as discussed in Section 2.2.2.

We perform Algorithm 1 with $\lambda_{k,t}^{0}=100,\eta_{k}=1$ to obtain the optimized ProMPs and perform Algorithm 2 for task execution. In each experiment, we randomly selected from three different modes, with via-points sampled using Gaussian distributions to ensure diversity in the task execution. We repeated the experiment 100 times to evaluate the performance for three modes; we ran the movement optimization and adaptation algorithm in parallel, with a total running time of 1.79664 s with a 100% success rate. The results are shown in Figure 3b,c.

The results demonstrate that we can efficiently generate feasible collision-free trajectory sets from only one human demonstration with via-point trajectory generalization. In addition, by encoding the joint positions with ProMPs, the discrete via-points can generate continuous distributions representing various skills for task execution as well as flexibility in performing the same skill. By applying different task constraints, new distributions tailored to specific tasks are derived. These distributions can be utilized directly for robotic task execution, allowing the robot to preserve the characteristics of human demonstrations while employing diverse skills to complete tasks effectively. In this experiment, we found that there were no feature timesteps violating task constraints. The reason is that waypoint constraints and hyperplane constraints are ensured by trajectory generalization, with the probability of satisfying constraints always becoming 1. For repeller constraints, the water bottle height is not high, so robot link 6 to link 1 will not collide if link 7 is safe. Meanwhile, for the above and left modes, if robot link 7 is safe, robot link 6 to link 1 will not collide, considering the robot configuration. As a result, to further evaluate the proposed method, we increase the task difficulty by elevating the obstacle’s height, restricting the robot to move right around the obstacle and extending the scenario to multiple obstacles.

### 4.2. Experiments with Various Obstacles

**Figure 4:**
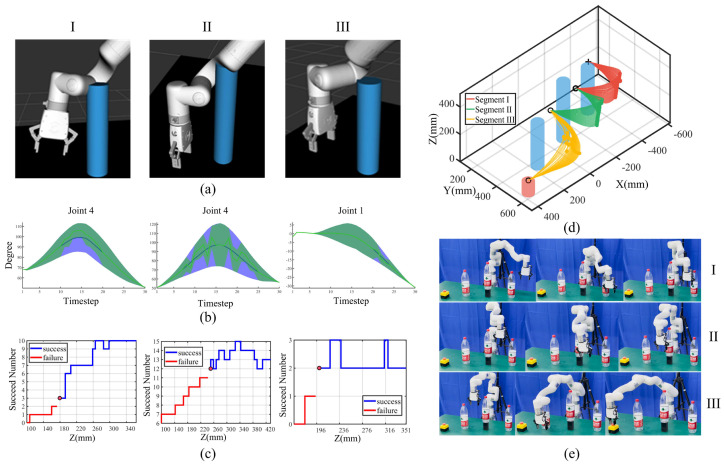
Experiments in a dangerous scene. I, II, and III denote three trajectory segments. (**a**) Dangerous cases shown in the ROS rviz, with blue cylinders denoting obstacles. (**b**) Typical original (blue) and optimized (green) ProMPs under task constraints. Joint mean values (2x standard deviation) are denoted by thick lines (shaded areas). (**c**) Threshold determination results. Blue and red lines are Z-positions leading to success and failure, with red points denoting the threshold. (**d**) Task execution trajectories for three segments, with red, green, and yellow lines (points) denoting generalized trajectories (feature via-points). (**e**) Robot task execution snapshots. The first, middle, and last rows denote I, II, and III trajectory segments.

To test the performance of the proposed task-constrained movement adaptation algorithm in critical scenarios involving task constraint violation and multiple obstacles, we set a dangerous scene where three high obstacles exist, and the robot was limited to moving right around the obstacle. We set proper local start and end points with feature via-points so that partial collision-free trajectory sets of link 7 would evidently collide at other links. To explore and validate the robot’s ability to adapt movements under these conditions, the entire trajectory was segmented into three segments. For each segment, we fixed the X- and Y-coordinates of the feature via-points and decreased the Z-coordinate, ensuring that other robot links would collide with the obstacle, as shown in the ROS Rviz simulation (Figure 4a). We defined the same task constraints as in the single-obstacle scenario in Section 4.1 for three segments, with the distinction that certain timesteps would violate task constraints and potentially cause collisions with robot links 4, 5, and 6.

For the three segments, after via-point trajectory generalization for the template trajectory, we obtain feasible collision-free trajectory sets of robot link 7 and encode the initial 7-DoF joint ProMP distribution. This distribution is then converted into Gaussian means and standard deviations for each timestep. Utilizing the moment-matching method, we determine the feature timesteps at which robot links 4, 5, and 6 will potentially collide with obstacles. We estimate the task constraint probability for these timesteps and incorporate task constraints, including joint constraints, hyperplane constraints, waypoint constraints, and repeller constraints, into the unified probabilistic framework. Utilizing double-loop LBFGS and EMM optimization in Algorithm 1, we obtain the optimized ProMPs satisfying task constraints, as shown in Figure 4b. The optimized joint distributions represent the feasible joint range for the robot to execute the task, which can be used as a reference for the Jacobian inverse kinematics solver.

However, considering the probabilistic nature of ProMPs, some distributions satisfying task constraints may be excluded during optimization. To refine this, we further set a threshold for the feasible joint range. Specifically, we allow the robot to generate initial collision-free trajectories from via-point trajectory generalization, obtaining the corresponding joint inverse kinematics solutions for each timestep. We then determine how many of these timesteps’ values fall within the optimized joint range. A threshold is set such that if the number of valid timesteps exceeds this threshold, the robot proceeds with executing the trajectory; otherwise, the robot stops.

For threshold determination, the robot automatically selects the same via-point range as in the initial generalization process and records the number of valid timesteps within the joint range and the corresponding obstacle avoidance results. A threshold is then established, where the robot executing the tasks is considered successful if the number of valid timesteps exceeds the threshold and is considered to have failed if it falls below. The threshold determination results are shown in Figure 4c, and the robot task execution results are shown in Figure 4d,e.

There are a total of 10, 15, and 3 feature timesteps violating repeller constraints, with the thresholds determined to be 3, 12, and 2 in the three trajectory segments. Running Algorithm 2 on segments I, II, and III in parallel results in a total time of 315.07 s. There are 71, 54, and 58 trajectories executed from 100 feature via-points in the three segments, and all executed trajectories satisfy task constraints with a 100% success rate.

The results illustrate that the robot can utilize sparse and discrete feature via-points to encode the initial ProMPs and optimize the ProMPs by integrating all task constraints into a probabilistic framework analytically. Due to the probabilistic nature of the approach, some distributions that satisfy task constraints may be excluded during optimization. Therefore, it is essential to further refine the feasible joint range by determining a threshold. This process is automated and leverages the same discrete and sparse via-point range used during trajectory generalization. By adding an additional threshold for the optimized joint range, the robot can derive feasible joint trajectories for task execution across a continuous range using only a discrete set of via-points. Specifically, the robot generates generalized trajectories, computes the corresponding joint values, and determines how many timesteps’ joint values fall within the optimized range given the threshold. These results are then applied directly to task execution, allowing the robot to effectively distinguish between success and failure scenarios. In a word, the threshold and optimized ProMPs are directly used for task execution by sampling from continuous Gaussian distributions. We thus provide the robot with the ability to perform tasks in a generic way; that is, there is no need for the robot to determine whether task constraints are satisfied every time during task execution.

**Figure 5:**
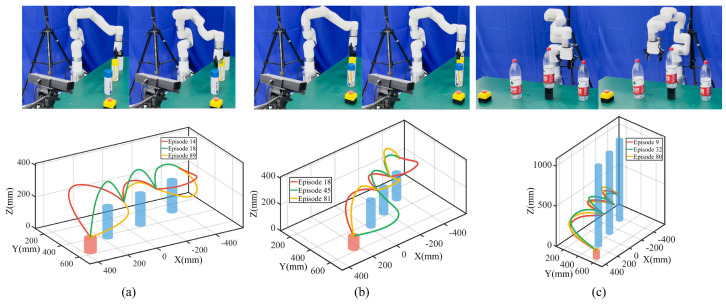
Experiments with different relative positions and nearly infinitely high obstacles. The gripper is (**a**) between and (**b**) above two obstacles. (**c**) Case with nearly infinitely high obstacles. The first rows denote local start and end points, and the second rows show typical trajectories colored in red, yellow, and green.

Furthermore, we set different relative obstacle positions and set the obstacle height to be nearly infinite. We chose three spray paint cans with different colors in two cases, where the gripper is between and above two adjacent obstacles, and set the water bottle height to 1000 mm. We repeated 100 experiments and recorded the total time as 3.25874 s, 2.45945 s, and 0.87882 s, with the results shown in Figure 5. For different relative obstacle positions, there are no timesteps violating task constraints with the trajectory directly utilized for task execution sampling from three modes. For nearly infinitely high obstacles, robot links 6, 5, and 4 will always collide at some timesteps, and the probability of satisfying task constraints always becomes 0. We thus exclude those modes, retaining only the remaining mode, and all experiments exhibit a 100% success rate.

The results show that the robot can autonomously select local generalization points facing different relative obstacle positions and exclude those modes that cannot satisfy task constraints, thereby achieving flexible movement adaptation. The robot’s diverse task execution skills play a critical role in this process. When a single skill is insufficient to complete the task, the robot can leverage alternative skills to optimize and adapt. Furthermore, if all available skills fail to meet the task constraints, human demonstrations are introduced to offer additional guidance, facilitating iterative demonstration and optimization to tackle these challenges.

### 4.3. Comparison Experiments

#### 4.3.1. Via-Point Trajectory Generalization Performance

Aiming to evaluate the via-point trajectory generalization performance, we compared two processes: template trajectory generation from human demonstrations with no via-points (see Figure 2) and initial collision-free trajectory set generalization with feature via-points (see Figure 3). We compared our method to DMPs [12] without via-points and VMPs [16] with via-points. For template trajectory generation, considering there are no via-points, the trajectories generalized with VMPs are the same as in our method; we thus selected DMPs for the comparison. For initial collision-free set generalization, considering that DMPs cannot handle intermediate via-points, we selected VMPs for the comparison.

**Figure 6:**
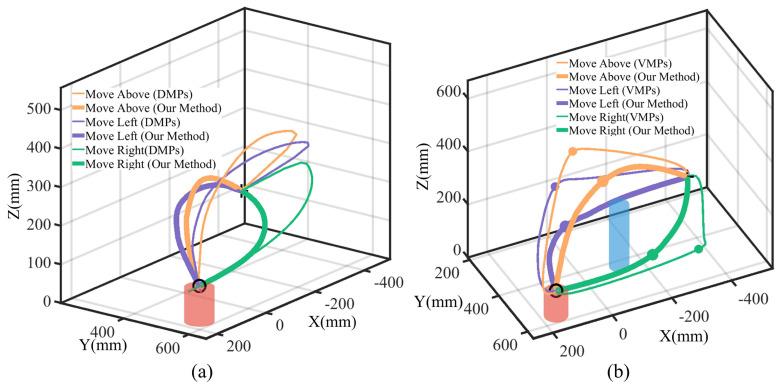
Via-point trajectory generalization comparison. (**a**) Comparison between DMPs and our method for template trajectory generation. Trajectory pairs in the same mode are depicted with lines with different widths. (**b**) Comparison between VMPs and our method for collision-free trajectory generalization. Trajectories and via-points in the same mode are depicted with different widths.

For template trajectory generation, we selected the single-obstacle task scenario in Section 4.1 and generated template trajectories utilizing both our method and DMPs, with visualized trajectories shown in Figure 6a. The results illustrate that our method preserves the human demonstration characteristics even when the task start and end points change. However, DMPs will lose demonstration information; the trajectories attached to the above and left modes will be generalized to the right mode. The reason is that DMPs are sensitive to the relative positions of the start and end points, leading to trajectories that deviate significantly from the demonstration.

Further, we designed experiments with five task conditions by varying the start and end points, making their relative distances progressively larger. For each task condition, we varied the start and end points across 20 distinct configurations. To quantitatively evaluate the similarity between generated trajectories and demonstrations, we adopted the cosine similarity measure by computing between each trajectory segment as

(5) $$M\_similarity=\frac{1}{T-1}\sum_{t=1}^{T-1}\frac{\left(x_{t+1}-x_{t}\right).\left(x_{t+1}^{*}-x_{t}^{*}\right)}{∥x_{t+1}-x_{t}∥∥x_{t+1}^{*}-x_{t}^{*}∥}$$

where M_similarity denotes the calculated trajectory similarity, $x_{t}$ and $x_{t}^{*}$ denote the generalized and demonstration trajectory vectors, and *T* is the total timesteps. This metric approximates the cosine similarity between two vectors, where values closer to 1 indicate higher similarity. Table 2 shows the trajectory similarity measure results.

From Table 2, we observe that when the positional offset between the start and end points from the demonstration is small, the trajectory similarity metrics for DMPs and our method are comparable. In such cases, DMPs retain most features of the human demonstration. However, as the positional offset increases, the trajectory similarity metric for our method remains consistently high, while that for DMPs declines rapidly. This demonstrates that our method is less affected by changes in the task’s initial and terminal conditions, adapting to varying task conditions.

For initial collision-free trajectory generalization with via-points, given the template trajectory, we evaluated the performance across different task scenarios, including a single obstacle (See Figure 3) and various obstacles in three segments: I, II, and III (see Figure 4). We selected representative trajectories for visualization, with the results shown in Figure 6b. We observe that VMPs produce unnatural transitions at via-points, generating discontinuous trajectories and causing significant interruptions at the via-points, which deviate from the characteristics of human demonstrations. To quantitatively measure trajectory smoothness, we calculate the derivation of the acceleration as follows:

(6) $$M\_smoothness=\frac{1}{T-3}\sum_{t=1}^{T-3}∥{\dddot{x}}_{t}∥$$

where M_smoothness denotes the calculated trajectory smoothness, and ${\dddot{x}}_{t}$ is the acceleration derivation. A trajectory smoothness closer to 0 indicates smoother trajectories. We evaluated the smoothness in four task scenarios, with the results summarized in Table 3. The results indicate that our method consistently achieves similar smoothness across different scenarios. In contrast, the smoothness for VMPs is relatively low in the single-obstacle case but increases significantly in the multi-obstacle scenarios. Compared to our method, VMPs exhibit less smooth transitions.

Comparative experiments demonstrate that our approach can generate template trajectories adapted to varying task conditions from a single demonstration. It effectively preserves the key characteristics of the human demonstration while utilizing via-point generalization to produce smooth and collision-free initial trajectory sets. These trajectories enable smooth transitions across different task scenarios, offering high-quality initial datasets for subsequent optimization processes.

#### 4.3.2. Task-Constrained Movement Adaptation Performance

In this paper, we propose Algorithms 1 and 2 for robot task-constrained movement adaptation. Algorithm 1 starts with an initial ProMP distribution, converts it into Gaussian distributions for each timestep, and determines timesteps violating task constraints in advance for optimization. The optimized Gaussian distributions for each timestep are then optimized again to obtain the optimized ProMP distributions. Algorithm 2 introduces an additional threshold as a reference for the optimized joint range of the ProMPs, enabling the robot to execute tasks directly without the need for repeated task constraint verification. This approach can also be extended to multi-obstacle scenarios, ensuring efficient task execution in more complex environments. To further evaluate and compare the robustness across different task scenarios, we conducted a comprehensive comparison with similar approaches in diverse task scenarios, including a single obstacle (see Figure 3), multiple hazardous obstacles (see Figure 4), infinitely high obstacles (see Figure 5c), and varying relative positions for multiple obstacles (see Figure 5a,b).

(1) Validation in Single-Obstacle Scenarios

Focusing on Algorithm 1, a similar approach is proposed in [22], where multiple skills are demonstrated through kinesthetic teaching and encoded as ProMP distributions. However, unlike our method, their approach directly optimizes the ProMP parameters by predefining all task constraints and estimating the distribution’s mean and covariance using an unscented transformation. The optimized ProMP distributions are then directly used by the robot to sample joint trajectories for task execution. In contrast, our method generalizes discrete via-points to generate continuous ProMP distributions and employs a threshold-based mechanism for task execution after optimizing the ProMP distribution.

To ensure a fair comparison, we used the same initial ProMP distribution for both methods. We then optimized the ProMP distribution using our approach and the method outlined in [22] and applied the resulting optimized distributions for task execution. In this comparison, we prioritized optimization performance over addressing movement adaptation in multi-obstacle environments. As a result, we first considered a single-obstacle case, as multi-obstacle tasks can be decomposed into sequential single-obstacle phases. Specifically, we designed four task scenarios: a single obstacle (see Figure 3) and three sequential segments (I, II, and III) with three high hazardous obstacles (see Figure 4). To assess the algorithm’s robustness across different task scenarios, for each scenario, we extensively varied obstacle positions and start and end points, resulting in a total of 30 test cases.

The robot’s task execution is evaluated using metrics such as the number of optimized parameters, the optimization time, the task success rate, and the loss between the initial and optimized distributions. Here, the loss between initial and optimized distributions refers to the overall proportions of the joint range across all timesteps after optimization compared to the original joint range. We record the metrics and summarize the results in Table 4.

Table 4 illustrates that, although our method optimizes more parameters overall compared to the approach in [22], it significantly reduces the optimization time. The reason is that we only optimize those timesteps violating task constraints. In the scenario with a single obstacle, at most timesteps, the Gaussian distributions already satisfy the task constraints, allowing our method to directly use the optimized distributions for task execution. In contrast, ref. [22] optimizes all ProMP parameters across every timestep, which can alter distributions that initially satisfied the task constraints. This over-optimization can lead to task failure, as the robotic arm may eliminate motions capable of completing the task. While the robot may still execute some tasks, the range of motion demonstrated during training is reduced.

To prevent the probabilistic optimization from excluding certain distributions, our method introduces an additional threshold to the optimized joint distributions and serves as a reference for the Jacobian inverse kinematics solver. When the number of inverse kinematics solutions exceeds this threshold, the robot successfully executes the trajectory. This threshold-based mechanism ensures that the robot retains useful information from the optimization process, allowing for more flexible skills to select from. Consequently, our method achieves reduced distribution loss with better utilization of demonstrated skills.

In segments I, II, and III for three high obstacles, ref. [22] tends to lose significant information during optimization, leading to task failures, especially when moving obstacles on the right side. Our method remains successful in task execution, even in high-risk conditions, producing more feasible motions as the difficulty of the scenario increases. Note that our method may fail in edge cases where the threshold intersects the boundary of the continuous space. In other words, once the threshold is defined, trajectories near it may initially be executed, and after the robot executes them, failures will occur. Increasing the via-point number or sacrificing certain distributions may overcome this drawback and improve task success rates.

(2) Validation in Multi-Obstacle Scenarios

We also evaluated our method in multi-obstacle scenarios, comparing it to off-the-shelf robotic movement adaptation algorithms like the sampling-based RRT-Connect [30] and probability-based STOMP [31]. Focusing on multiple hazardous obstacles (see Figure 4), infinitely high obstacles (see Figure 5c), and varying relative positions for multiple obstacles (see Figure 5a,b), we changed the relative positions of the obstacles across 30 configurations in each scenario.

**Figure 7:**
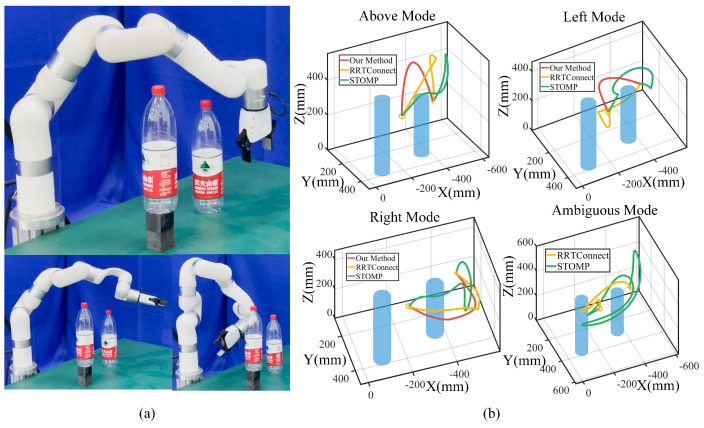
Movement adaptation comparison. (**a**) Snapshots of task execution poses. The first row shows our method, and the second row shows STOMP and RRTConnect. (**b**) Different mode trajectories. Trajectories generated by Algorithm 2, RRTConnect, and STOMP are denoted in red, yellow, and green. Trajectories not belonging to any modes are treated as ambiguous modes.

For visualization, a representative trajectory is shown in Figure 7, where the same start and end points are used to compare the performance of our method with that of STOMP and RRT-Connect. The generated trajectories are classified based on predefined categories, with the results shown in Figure 7. The robot randomly selects from three modes with our method, while the trajectories for RRT-Connect and STOMP are recorded and categorized into three modes. If those trajectories do not belong to any of the three modes, we record them separately and define them as ambiguous modes. We discover that the RRT-Connect and STOMP trajectories are disorganized and unstable, and sometimes, the robot takes a meandering trajectory (see Figure 7b), leading to large deviations between adjacent timesteps.

To quantitatively evaluate the performance, we selected several metrics to test the aforementioned task scenarios, where the relative positions of the obstacles varied randomly. We define the normalized sum of pose errors ΔP between two adjacent timesteps as $\Delta P=\sum_{i=1}^{T-1}\left|q_{i+1}-q_{i}\right|$ and the modulated path L as $L=\sum_{i=1}^{T-1}\left|p_{i+1}-p_{i}\right|$, with the corresponding energy efficiency defined as $\eta =\frac{L_{\min}}{L}.\frac{\Delta P_{\min}}{\Delta P}$, where $L_{\min}$ and $\Delta P_{\min}$ denote the minimum path length and amplitude. The closer the energy efficiency to 1, the higher the efficiency of the robot in performing the task. We also evaluated robustness across different task scenarios, measured by the task success rate. Additionally, since our method involves offline optimization, followed by online execution, we calculated both offline optimization time and online execution time. The results are presented in Table 5.

The results demonstrate that our method generates shorter modulated paths but requires considerable offline planning time. This can be attributed to the fact that our approach fully incorporates task constraints, thereby representing multiple feasible skills to accomplish the task. After optimization, we directly execute the trajectories with shorter online execution times. STOMP and RRT-Connect calculate and sample a single trajectory for execution without considering multiple task execution skills. They exhibit significant randomness, sometimes taking unnecessarily long detours to complete the task when a direct route will suffice. While imposing additional constraints could reduce such randomness, the robot’s behavior often lacks consistency and continuity. In contrast, our method maintains trajectory continuity with smaller variations in posture, facilitating smoother transitions and reduced energy consumption.

Meanwhile, our method exhibits a higher success rate in scenarios involving infinitely high obstacles, the reason being that STOMP and RRT-Connect often struggle to find successful trajectories due to the infinite height of the obstacles. In some instances, they fail midway through the task or become trapped in local minima when approaching high obstacles. In contrast, our method is capable of excluding infeasible skills and selecting alternative skills to complete the task, allowing for more flexible and adaptive movements. However, in critical situations where via-point generalization cannot generate effective collision-free trajectories, our method is prone to failure.

## 5. Conclusions

### 5.1. Significance

This paper proposes a robot task-constrained optimization and adaptation method with ProMPs. The method allows the robot to adapt its movements under various task constraints and extend to multiple obstacles, focusing on task execution flexibility with different skills. We present a via-point trajectory generalization method that learns from only one human demonstration for each skill category. This method effectively adapts to varying task conditions while preserving the key characteristics of the human demonstration. The discrete via-points generate continuous distributions encoded with ProMPs, representing various skills for task execution as well as flexibility in performing the same skill.

Given initial ProMPs, we incorporate all task constraints into a unified probabilistic framework, decouple ProMPs as Gaussian distributions at each timestep, and only optimize those timesteps violating task constraints, significantly reducing the optimization time. To prevent the probabilistic optimization from excluding certain distributions, our method introduces an additional threshold to the optimized joint distributions, serving as a reference for the Jacobian inverse kinematics solver. When the number of inverse kinematics solutions exceeds this threshold, the robot successfully executes the trajectory. This threshold-based mechanism ensures that the robot retains useful information from the optimization process, allowing for more flexible skill selection.

Meanwhile, we propose a movement adaptation method for multiple obstacles, enabling the robot to autonomously select local generalization points based on different relative obstacle positions and exclude modes that cannot satisfy task constraints. When a single skill is insufficient to complete the task, the robot can leverage alternative skills for optimization and adaptation. Furthermore, if all available skills fail to meet the task constraints, human demonstrations are introduced to provide additional guidance, facilitating iterative demonstration and optimization to tackle these challenges.

### 5.2. Limitations

However, our approach has certain limitations and may fail in specific scenarios. It relies on the quality of initial collision-free end-effector trajectory sets. In confined spaces with multiple obstacles, it is challenging to generalize trajectories to generate a substantial collection of collision-free trajectories. Consequently, the subsequent optimization lacks a high-quality dataset, making it difficult for the robot to optimize the distribution capable of completing the task with a limited dataset.

Additionally, we define thresholds using discrete via-points. If the selected via-points are too sparse, it becomes difficult to represent the distribution in continuous space. At the extreme threshold positions, the optimization results might suggest that the robotic arm can perform the task, but the actual task execution often fails. Moreover, even if the robotic arm generates a large initial set of collision-free trajectories, it may still fail to satisfy all task constraints despite optimization. This is particularly evident when encountering obstacles of infinite height. While the end-effector may avoid collisions, the distributions of other joints might violate task constraints, leading to failure in selecting appropriate skills to complete the task.

Furthermore, our method is specifically tailored for simple pick-and-place tasks. The limited number of demonstrations may impact generalizability in complex tasks requiring diverse scenarios. As a result, for more complex tasks involving object manipulation, a finer-grained via-point generalization method is necessary to generate high-quality datasets for subsequent optimization. In cases with multiple obstacles, our approach determines local start and end points based on relative positions. However, more complex obstacle distributions demand a more heuristic motion pattern switching strategy to ensure task success. Meanwhile, our method cannot handle multiple real-time dynamic obstacles.

### 5.3. Future Work

To address the aforementioned limitations, future work should focus on more complex object manipulation tasks, exploring methods for generating high-quality initial feasible motion sets from human demonstrations. This includes improving the via-point generalization method and incorporating movement segmentation techniques to divide the movement into multiple trajectory segments, each of which will be generalized and combined to generate high-quality datasets. Additionally, further exploration is needed for threshold determination processes, ensuring that the discrete via-points better align with the distribution of continuous space. Moreover, future research should explore biologically inspired motion-mode-switching techniques, such as the gradient-free control approach derived from the natural behavior of beetles for simultaneous static and dynamic obstacle avoidance, as outlined in [32]. Integrating this method with our task constraints could enhance its suitability for real-time applications.

## Figures and Tables

**Table 1:** Definitions and notations in this paper.

**Dimensions**	**Description**
*T*	Timesteps of a trajectory
*n*	The window width of the Moving Average Filter (MAF)
*N*	The demonstration number
*D*	The DoF number of the robot
*K*	The number of Gaussian basis functions
**Feature Spaces**	**Description**
$p=\left[x,y,z\right],\;o=\left[q_{w},q_{x},q_{y},q_{z}\right]$	Position p and orientation o sets of the human hand centroid
$y\left(p\right),\;y\left(o\right)$	Combined 3D and orientation trajectories
$h\left(p\right),\;h\left(o\right)$	Elementary 3D and orientation trajectories
$f\left(p\right),\;f\left(o\right)$	3D and orientation shape modulation
$p_{1},\;p_{end}$	The start and end 3D points
r	The 3D scale factor denoting the proportion of projective points
$t_{\mathrm{via}},\;p_{\mathrm{via}}$	The timestep and 3D positions of a via-point
*k*	The 3D shape modulation coefficient
${\left\{q_{t}\right\}}_{i=1}^{N}$	All joint sets of the robot with the inverse kinematics (IK) algorithm
$q_{k,t}^{\min},\;q_{k,t}^{\max}$	The minimum and maximum joint positions for *k*-th joint’s freedom
${\bar{X}}_{t}$	The expected waypoint positions
$n_{k,t},\;b_{k,t}$	The hyperplane normal and bias vectors
$d_{\min}$	The minimum distance between the robot and repellers
$R_{\mathrm{safe}}$	The repeller safety radius
**Distributions**	**Description**
$\Psi \left(t\right)$	Gaussian basis functions
$w_{i}$	The weight vector of the *i*-th Gaussian kernel
$\varepsilon_{n}$	Zero-mean Gaussian noise
$\left\{\mu_{w},\Sigma_{w}\right\}$	The mean and covariance of the weight vector
$\Sigma_{n}$	The Gaussian noise covariance
$p_{0}\left(w\right)=N\left(w;\mu_{w}^{0},\Sigma_{w}^{0}\right)$	Initial Gaussian distributions of ProMPs
$p^{*}\left(w\right)=N\left(w;\mu_{w}^{*},\Sigma_{w}^{*}\right)$	Optimized Gaussian distributions of ProMPs
**Distributions**	**Description**
$\theta_{0}=\left(\mu_{t}^{0},\sigma_{t}^{0}\right)$	The initial mean and standard deviation of each timestep
$\theta^{*}=\left(\mu_{t}^{*},\sigma_{t}^{*}\right)$	The optimized mean and standard deviation of each timestep
$c_{k,t}=C\left(\mu_{t},\sigma_{t}\right)$	The *k*-th task constraint function related to $\left(\mu_{t},\sigma_{t}\right)$
$F_{c_{k,t}}\left(\mu_{t},\sigma_{t}\right)$	The cumulative distribution function (CDF) of the *k*-th task constraints
$H\left(c_{k,t}\right)$	One-sided or two-sided inequality constraints
$P_{\mu_{t},\sigma_{t}}\left(H\left(c_{k,t}\right)\right)$	Task constraint probability
**Distributions**	**Description**
$\alpha_{k,t}$	The confidence level to represent the task constraint probability
$d_{k,t}$	The distance threshold to represent the task constraint probability
$S=\{c_{k,t}\left(q_{1},q_{2},...,q_{D}\right),\;q_{k}\in \left\{\mu_{k}-2\sigma_{k},\mu_{k}+2\sigma_{k}\right\}\}$	The estimation set for nonlinear task constraints
$E\left(c_{k,t}\right),V\left(c_{k,t}\right)$	The estimated mean and variance of nonlinear task constraints
$\Gamma \left(\alpha ,\beta \right)$	A Gamma distribution with shape α and rate β
$D_{\mathrm{KL}}\left[\theta ||\theta_{0}\right]$	The KL divergence between two univariate Gaussian distributions
$\lambda_{k,t}$	The Lagrange multiplier
$L\left(\theta ,\left\{\lambda_{k,t}\right\}\right)$	The Lagrangian function
$\eta_{k}$	The convergence speed parameter

**Table 2:** Trajectory similarity measurement results for DMPs and our method (the relative distance r represents the normalized relative distances observed during the experiment). For each task condition, we varied the start and end points across 20 distinct configurations.

Trajectory Similarity	Task 1 (r = 0–0.2)	Task 2 (r = 0.2–0.4)	Task 3 (r = 0.4–0.6)	Task 4 (r = 0.6–0.8)	Task 5 (r = 0.8–1)
DMPs	0.9563 ± 0.1064	0.8973 ± 0.1564	0.7654 ± 0.1318	0.6424 ± 0.1389	0.5575 ± 0.2289
Our method	0.9845 ± 0.0184	0.9732 ± 0.0264	0.9667 ± 0.0187	0.9674 ± 0.0204	0.9574 ± 0.0584

**Table 3:** Trajectory smoothness results for VMPs and our method. Given the template trajectory, we evaluated the performance across different task scenarios, including a single obstacle (see Figure 3) and various obstacles in three segments: I, II, and III (see Figure 4).

Trajectory Smoothness	A Single Obstacle	Various Obstacles		
		Segment I	Segment II	Segment III
VMPs	0.1043 ± 0.0275	0.1843 ± 0.0395	0.2864 ± 0.0286	0.3365 ± 0.0489
Our method	0.0752 ± 0.0146	0.0864 ± 0.0123	0.0796 ± 0.0452	0.0852 ± 0.0204

**Table 4:** Comparisons with single-obstacle scenarios. We designed four task scenarios: one involving a single obstacle (see Figure 3) and three sequential segments (I, II, and III) with three high hazardous obstacles (see Figure 4). Each scenario involves 30 variations of task conditions.

Task Scenario	Method	Optimized Parameters	Optimized Time	Success Rate	Distribution Loss
A single obstacle	Algorithm in [22]	665	20.4586 s ± 4.2367 s	96.67%	85.46% ± 3.24%
	Our method	679 ± 14	1.7904 s ± 0.1986 s	100%	98.74% ± 0.16%
Segment I	Algorithm in [22]	665	1204.32 s ± 48.52 s	63.33%	64.72% ± 5.79%
	Our method	749 ± 56	346.08 s ± 86.34 s	93.33%	85.48% ± 3.56%
Segment II	Algorithm in [22]	665	1365.38 s ± 49.37 s	73.33%	62.62% ± 4.28%
	Our method	805 ± 70	479.28 s ± 65.28 s	90.00%	79.49% ± 4.68%
Segment III	Algorithm in [22]	665	864.37 s ± 32.58 s	80.00%	73.52% ± 5.62%
	Our method	707 ± 42	273.58 s ± 57.42 s	96.67%	89.37% ± 3.56%

**Table 5:** Comparisons in multi-obstacle scenarios. For multiple hazardous obstacles (see Figure 4), infinitely high obstacles (see Figure 5c), and varying relative positions for multiple obstacles (see Figure 5a,b), we changed the relative positions of the obstacles across 30 configurations in each scenario.

Task Scenario	Method	Pose Errors	Path Length	Energy Efficiency	Success Rate	Offline Time	Online Time
Hazardous	STOMP	0.81 ± 0.19	10.37 m ± 1.52 m	63.25% ± 5.76%	96.67%	0	0.063 s ± 0.023 s
	RRT-Connect	0.87 ± 0.13	11.36 m ± 2.34 m	59.25% ± 6.38%	100%	0	0.054 s ± 0.068 s
	Our method	0.15 ± 0.05	0.82 m ± 0.03 m	84.62% ± 2.34%	96.67%	304.2 s ± 42.4 s	0.015 s ± 0.004 s
Infinitely High	STOMP	0.75 ± 0.14	6.72 m ± 0.96 m	68.64% ± 3.28%	83.33%	0	0.123 s ± 0.032 s
	RRT-Connect	0.82 ± 0.25	7.48 m ± 2.31 m	67.24% ± 2.48%	80.00%	0	0.086 s ± 0.029 s
	Our method	0.22 ± 0.12	0.52 m ± 0.04 m	86.26% ± 1.34%	93.33%	2.83 s ± 0.65 s	0.017 s ± 0.006 s
Varying	STOMP	0.76 ± 0.13	9.52 m ± 1.46 m	56.72% ± 4.38%	100%	0	0.076 s ± 0.024 s
	RRT-Connect	0.68 ± 0.25	10.28 m ± 2.74 m	53.47% ± 2.42%	100%	0	0.046 s ± 0.013 s
	Our method	0.09 ± 0.02	0.42 m ± 0.02 m	93.25% ± 1.59%	100%	4.96 s ± 0.35 s	0.012 s ± 0.003 s

## Data Availability

The original contributions presented in the study are included in the article, further inquiries can be directed to the corresponding authors.
